# Liver and White/Brown Fat Dystrophy Associates with Gut Microbiota and Metabolomic Alterations in 3xTg Alzheimer’s Disease Mouse Model

**DOI:** 10.3390/metabo12040278

**Published:** 2022-03-22

**Authors:** Maria Angela Guzzardi, Federica La Rosa, Daniela Campani, Maria Carmen Collado, Daniel Monleon, Andrea Cacciato Insilla, Maria Tripodi, Alessandro Zega, Alessia Dattilo, Maurizia Rossana Brunetto, Margherita Maffei, Ferruccio Bonino, Patricia Iozzo

**Affiliations:** 1 Institute of Clinical Physiology, National Research Council (CNR), 56124 Pisa, Italy; larosa.fed@gmail.com (F.L.R.); maria.t@email.it (M.T.); alessandro.zega@ifc.cnr.it (A.Z.); m.maffei@ifc.cnr.it (M.M.); patricia.iozzo@ifc.cnr.it (P.I.); 2 Department of Surgical, Medical, Molecular Pathology and Critical Care Medicine, Division of Pathology, Pisa University Hospital, 56124 Pisa, Italy; daniela.campani@unipi.it (D.C.); andrea.cacciatoinsilla@gmail.com (A.C.I.); 3 Institute of Agrochemistry and Food Technology-National Research Council (IATA-CSIC), 46980 Valencia, Spain; mcolam@iata.csic.es; 4 Faculty of Medicine, Health Research Institute INCLIVA/CIBERFES for Frailty and Healthy Aging, University of Valencia, 46003 Valencia, Spain; daniel.monleon@uv.es; 5 Scuola Superiore Sant’Anna, 56127 Pisa, Italy; a.dattilo@santannapisa.it; 6 Department of Clinical and Experimental Medicine, University of Pisa, 56124 Pisa, Italy; maurizia.brunetto@unipi.it; 7 Hepatology Unit, Department of Medical Specialties, Laboratory of Molecular Genetics and Pathology of Hepatitis Viruses, Pisa University Hospital, 56124 Pisa, Italy; 8 Institute of Biostructure and Bioimaging (IBB), National Research Council (CNR), 80145 Napoli, Italy; ferruccio.bonino@unipi.it

**Keywords:** Alzheimer’s disease mouse model, liver inflammation, adipose tissues, gut microbiota, metabolome, PET-CT imaging

## Abstract

Metabolic impairments and liver and adipose depots alterations were reported in subjects with Alzheimer’s disease (AD), highlighting the role of the liver–adipose–tissue–brain axis in AD pathophysiology. The gut microbiota might play a modulating role. We investigated the alterations to the liver and white/brown adipose tissues (W/BAT) and their relationships with serum and gut metabolites and gut bacteria in a 3xTg mouse model during AD onset (adulthood) and progression (aging) and the impact of high-fat diet (HFD) and intranasal insulin (INI). Glucose metabolism (^18^FDG-PET), tissue radiodensity (CT), liver and W/BAT histology, BAT-thermogenic markers were analyzed. 16S-RNA sequencing and mass-spectrometry were performed in adult (8 months) and aged (14 months) 3xTg-AD mice with a high-fat or control diet. Generalized and HFD resistant deficiency of lipid accumulation in both liver and W/BAT, hypermetabolism in WAT (adulthood) and BAT (aging), abnormal cytokine–hormone profiles, and liver inflammation were observed in 3xTg mice; INI could antagonize all these alterations. Specific gut microbiota–metabolome profiles correlated with a significant disruption of the gut–microbiota–liver–adipose axis in AD mice. In conclusion, fat dystrophy in liver and adipose depots contributes to AD progression, and associates with altered profiles of the gut microbiota, which candidates as an appealing early target for preventive intervention.

## 1. Introduction

Altered adiposity is a pathogenic factor associated with Alzheimer’s disease (AD) [1]. It has been demonstrated that obesity and liver disease, including steatosis and inflammation, induce AD symptoms in wild-type animals and accelerate AD deterioration in transgenic models [2,3,4,5]. On the other hand, reduced body weight and deficiency of the adipocyte-derived hormone leptin have been observed in AD patients [6] and preclinical models [7]. Experimental evidence [7,8] suggests that beta-amyloid accumulation in AD could inhibit leptin-responsive hypothalamic neurons, causing alterations in the circuits devoted to body weight regulation and blunting the neuroprotective effects of the hormone [9]. The latter would result in a further loss of adipose tissue, leading to a vicious cycle worsening AD pathology. In the 3xTg AD mouse model, the reduction in peripheral leptin is associated with a reduction in cognitive function and cerebral glucose fractional extraction [2]. In addition, lower brown adipose tissue metabolism [10] and thermogenic deficits [11,12] have been reported in the transgenic Tg2576 and 3xTg AD mouse models. Overall, these findings highlight the role of the liver–adipose–tissue–brain axis in AD pathophysiology. However, existing data are controversial on the primacy of lipid accumulation, inflammation, and metabolic alterations in AD pathology.

Published evidence shows that intranasal insulin (INI) therapy improves cognitive deficits in animals [2,13,14] and patients [15], by restoring brain insulin signaling and modifying brain protein and inflammation [13,16]. The effects of intranasal insulin on the liver–adipose–tissue–brain axis [17,18] might contribute a therapeutic role in AD pathology.

In addition, the gut may play an important role in the above axis, given several associations reported between gut metabolome and microbiota modifications and obesity [19], type 2 diabetes [20], liver disease, and AD [21].

The aim of this study was to investigate the alterations occurring in liver and white and brown adipose tissues (WAT, BAT) during AD onset and progression, the contribution of a high-fat diet (HFD), and the relationships with serum and gut metabolites and bacteria in 3xTg male mice. The combined effect of a fatty diet and INI on cognitive decline and brain metabolism was previously published [2]. Here, in vivo positron emission tomography with ^18^F-labeled fluorodeoxyglucose (^18^FDG-PET) and computerized tomography (CT) were combined with histological tissue characterization and the analysis of thermogenic markers expression in BAT. Moreover, clinic–pathologic outcomes were also explored in relation to the gut microbiota and metabolome (serum, caecum, and colon).

## 2. Results

### 2.1. Glucose Metabolism

**Figure 1:**
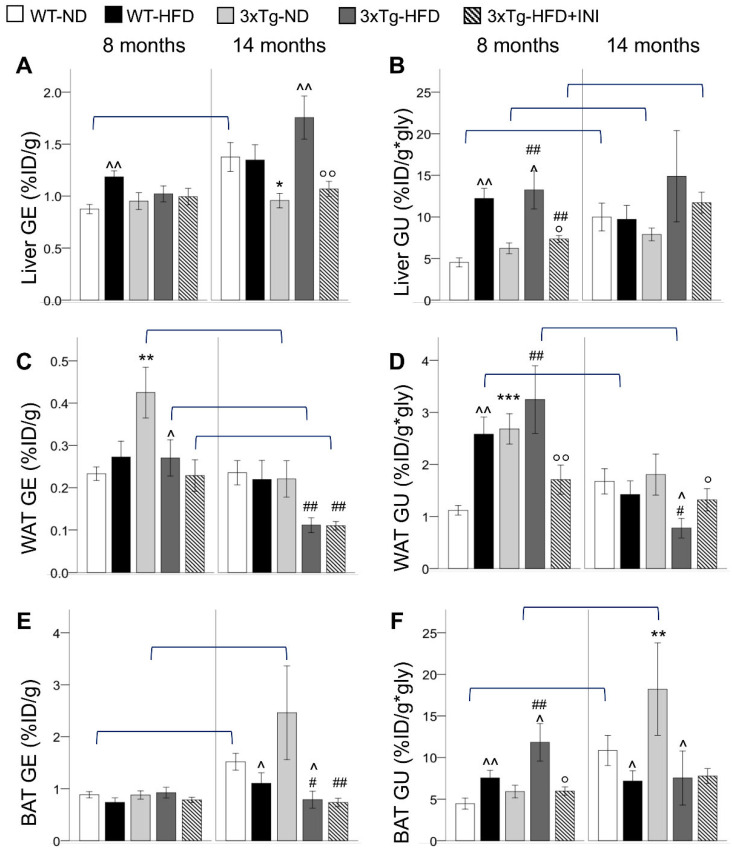
Fractional glucose extraction (GE) and glucose uptake (GU) in the liver (**A**,**B**), WAT (**C**,**D**), and BAT (**E**,**F**) in WT and 3xTg mice, fed ND or HFD, at age 8 and 14 months, are shown. Mean ± SEM shown. Different symbols are used for each within-age comparison, * *p* ≤ 0.05 vs. WT, ** *p* ≤ 0.01 vs. WT, *** *p* ≤ 0.001 vs. WT, ^ *p* ≤ 0.05 vs. ND, ^^ *p* ≤ 0.01 vs. ND, # *p* ≤ 0.05 vs. WT-ND, ## *p* ≤ 0.01 vs. WT-ND, ° *p* ≤ 0.05 vs. 3xTg-HFD, °° *p* ≤ 0.01 vs. 3xTg-HFD. A line is used to identify statistically significant differences in between-age comparisons.

**Figure 2:**
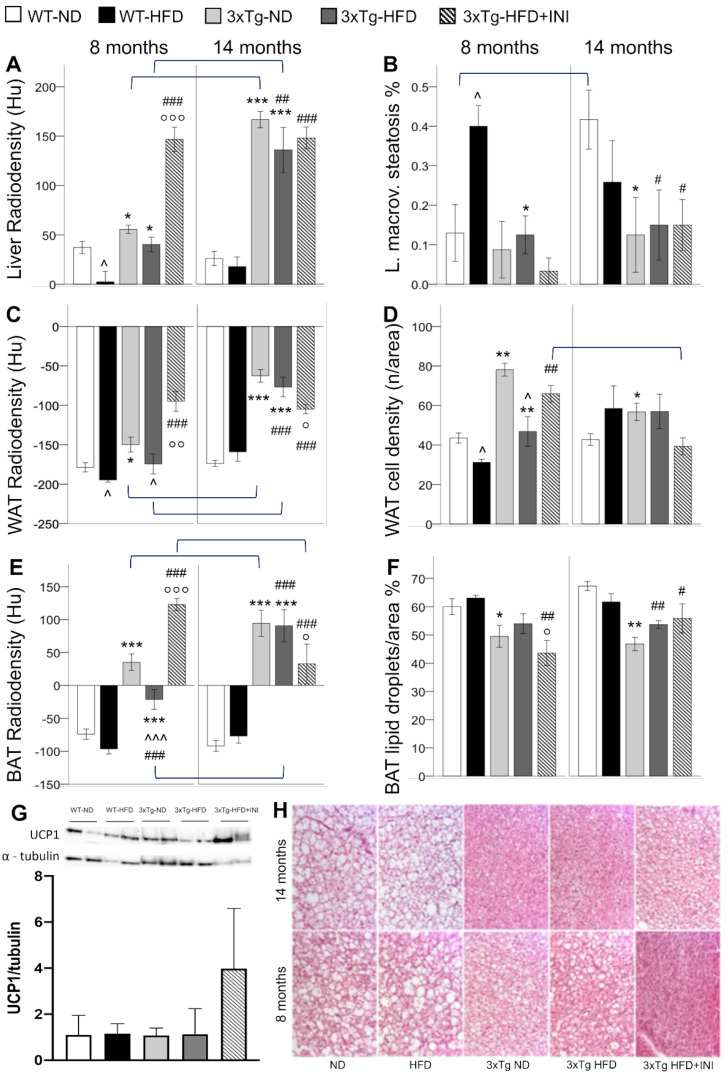
CT radiodensities of the liver, WAT, and BAT (**A**,**C**,**E**), and related histological features, i.e., liver macro-vesicular steatosis (**B**), WAT cell density (**D**), and BAT lipid droplets % (**F**) in WT and 3xTg mice, fed ND or HFD, at age 8 and 14 months. Bar graph shows the mean of two Western blot experiments for BAT UCP1 protein expression in 8 months old mice (**G**), and representative BAT histological sections H&E stained demonstrating the severe lipid depletion (white areas corresponding to lipid droplets) observed in 3xTg mice (**H**). Mean ± SEM shown. Different symbols are used for each within-age comparison, * *p* ≤ 0.05 vs. WT, ** *p* ≤ 0.01 vs. WT, *** *p* ≤ 0.001 vs. WT, ^ *p* ≤ 0.05 vs. ND, ^^^ *p* ≤ 0.001 vs. ND, # *p* ≤ 0.05 vs. WT-ND, ## *p* ≤ 0.01 vs. WT-ND, ### *p* ≤ 0.001 vs. WT-ND, ° *p* ≤ 0.05 vs. 3xTg-HFD, °° *p* ≤ 0.01 vs. 3xTg-HFD, °°° *p* ≤ 0.001 vs. 3xTg-HFD. A line is used to identify statistically significant differences in between-age comparisons.

During adult age, 3xTg mice fed normal diet (ND) showed higher GE and GU in WAT but not in BAT and liver, in which the glucose metabolism was comparable to wild-type (WT) ND mice (Figure 1). HFD increased the glucose extraction (GU) of all the three tissues in WT mice, in coherence with the elevation of fasting glycemia (Table 1). In 3xTg mice, HFD significantly increased GU in the liver and BAT, whereas in WAT, fractional glucose extraction (GE) was reduced, and the already high GU was not significantly incremented by the diet. Of note, the thermogenic UCP1 protein expression was not different between genotypes or diets (Figure 2G).

With aging, GE and GU increased in liver and BAT in ND-groups, whereas in WAT, HFD led to GU decrement, particularly in the 3xTg genotype in which an aging-related GE reduction was observed. As a result, in aged mice, GE and GU of 3xTg-ND compared to WT-ND mice were similar in WAT, slightly reduced in the liver, and greatly increased (around 2 folds) in BAT, showing significant hypermetabolism (Figure 1). HFD reduced the glucose metabolism of BAT in both mouse models, and of WAT in 3xTg mice only. Conversely, liver glucose extraction of the transgenic model was increased by the HFD.

Results suggest that in 3xTg compared to WT mice, WAT is a more efficient glucose sink, but this trait is lost under chronic exposure to HFD and with aging.

### 2.2. Tissue Lipid Content

CT imaging radiodensity is an in vivo marker inversely related to tissue lipid content, as proven by histological analyses. In the pooled population, significant correlations were found between lower liver radiodensity and greater macro-vesicular steatosis (r = −0.643, *p* < 0.0005), lower WAT radiodensity and lower cell density (r = 0.518, *p* < 0.0005), lower BAT radiodensity and higher percentage of lipid droplet per area (r = −0.791, *p* < 0.0005).

During adult age, the liver, WAT, and BAT radiodensity was significantly higher in 3xTg-ND compared to WT-ND (Figure 2). Consistently, higher cell density and lower lipid droplet % per unit area were histologically confirmed in WAT and BAT, respectively, and a trend for lower macro-vesicular and total steatosis was observed in the liver (Figure 2). HFD reduced radiodensity and cell density (i.e., increased lipid content) of WAT in both models. Radiodensity reduction was observed in BAT in 3xTg mice (a similar trend was found in WT mice), in which, however, lipid content remained significantly lower compared to WT mice (higher tissue radiodensity and trend of lower lipid droplets %), irrespective of dietary regimen. In the liver, HFD significantly reduced radiodensity in WT but not in 3xTg mice, as confirmed by the severe liver macro-vesicular steatosis observed in WT but not in 3xTg mice (Figure 2). Overall, data suggest that adult 3xTg mice are resistant to BAT whitening and fatty liver disease, even under HFD.

With aging, the radiodensity of the liver, WAT, and BAT was further increased in 3xTg mice but not in WT, resulting in significantly higher values in the 3xTg mice compared to controls, and only slightly reduced by HFD. Consistently, histological analysis revealed lower lipid droplets percentage in BAT and lower macrovesicular steatosis in the liver of 3xTg mice compared to WT under ND, and no significant change due to HFD (Figure 2). BAT CT and histology data are suggestive of an adipose tissue depot that has lost the storage function.

### 2.3. Liver Inflammation

**Figure 3:**
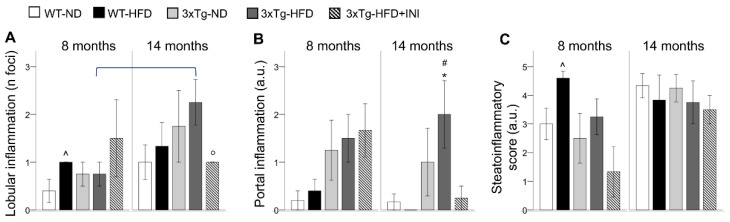
Liver lobular (**A**), and portal inflammation (**B**), and the cumulative steato-inflammatory score (**C**) are shown for each group. Mean ± SEM shown. Different symbols are used for each within-age comparison, * vs. WT, ^ vs. ND, # vs. WT-ND, ° vs. 3xTg-HFD. A line is used to identify statistically significant differences in between-age comparisons.

Altered liver metabolism (increased by HFD and aging) and lipid storage capacity (greatly reduced by aging) in 3xTg mice were reflected by a progressive increment in lobular and portal inflammation moving from WT-ND to 3xTg-HFD mice (Figure 3A,B), evident already from adult age (Figure 3B). In both genotypes, hepatocyte ballooning was observed only under HFD (data not shown). Fibrosis and hyper-glycogenic nuclei were absent in all the animals.

The cumulative steato-inflammatory score was significantly higher in WT-HFD mice, consistent with the 4-fold increment in macro-vesicular steatosis observed in these groups compared to the others (Figure 2B). An increasing trend was found also in adult 3xTg mice under HFD compared to their ND counterpart. With aging, between-group differences disappeared.

### 2.4. Circulating Markers

The 3xTg genotype was characterized by a dramatic depletion of circulating leptin, IL-6, and MCP-1 levels, and higher levels of PAI-1 compared to the WT genotype [2]. AST circulating levels were higher in 3xTg mice compared to their WT counterpart in the adult but not during older age (Table 1), whereas ALT was reduced in adult 3xTg-HFD mice compared to their WT counterpart. HFD increased triglyceride levels in both models (significantly in WT and showing a trend in 3xTg) at 8 but not at 14 months. An aging-related increment in body weight was observed in all groups (full statistical significance not reached in 3xTg-HFD only) and was accompanied by an increment in leptin in all groups, with the exception of the 3xTg-HFD mice in which circulating leptin did not show a further increment. With aging, circulating glucose and TG decreased in HFD mice reaching the levels observed in the ND genotype-matched mice. Moreover, an aging-related increment in MCP-1 and resistin was observed in the WT but not in the 3xTg group, irrespective of their dietary regimen.

Correlative analysis in the pooled population showed that plasma triglyceride levels were positively associated with tissue lipid accumulation (lower tissues radiodensity, higher BAT lipid droplets, higher liver steatosis at both ages, and lower WAT cell density, higher liver GU, ballooning, and total damage at 8 months only) (Table 2). However, at 8 months only, higher plasma triglycerides seemed protective against portal inflammation (negative correlation).

Leptin depletion was significantly correlated with higher organ radiodensity, higher WAT cell density, lower BAT lipid droplet %, lower macro-vesicular and total steatosis, and higher liver portal inflammation at both 8 and 14 months of age. At 14 months, low circulating IL-6, MCP-1 and leptin were related to high organs radiodensity, and to low liver GE. High PAI-1 was associated with high liver and BAT radiodensity, low liver steatosis, BAT lipid droplets, and high WAT cell density. At this age, but not at 8 months, tissues radiodensity was also negatively related to circulating resistin and insulin levels (Table 2). Overall, the correlations suggest that in 3xTg mice low leptin, IL-6, and MCP-1 and high PAI-1 predict low organ lipid accumulation and high liver portal inflammation.

### 2.5. Effect of Intranasal Insulin Therapy in 3xTg HFD Mice

INI administration prevented glucose hypermetabolism in WAT, BAT, and liver during adult age, consistent with the previously observed reduction in circulating glucose levels (Table 1). Moreover, INI reduced tissue lipid storage in all three organs, as documented by higher radiodensity values, higher WAT cell density, lower BAT lipid droplets, and lower liver steatosis.

In 3xTg-HFD-INI mice, aging led to a significant increment in liver GU, similar to the increment observed in ND mice, and a reduction in WAT cell density. In aged mice, INI administration normalized glucose hypometabolism of WAT and improved lipid storage capacity of WAT and BAT (lower radiodensity), which, however, remained significantly lower than in WT control mice. INI administration was protective against portal and lobular inflammation, which showed values similar to those observed in WT-ND controls (Figure 3). In addition, INI administration led to a 3-fold increment in BAT UCP1 protein expression at 8 months compared to 3xTg-HFD (Figure 2G). All these effects were accompanied by a significant increment in circulating IL-6 and MCP1 at 8 months, but a reduction in the HFD-dependent increment in IL-6 and resistin in aged 3xTg mice (Table 1).

Overall, INI administration favored the restoring of metabolic and morphological parameters of 3xTg-HFD mice towards those observed in the WT-ND mice, counteracting both the effect of HFD and the metabolic impairments associated with the genetic 3xTg background.

### 2.6. Serum and Fecal Metabolome

**Figure 4:**
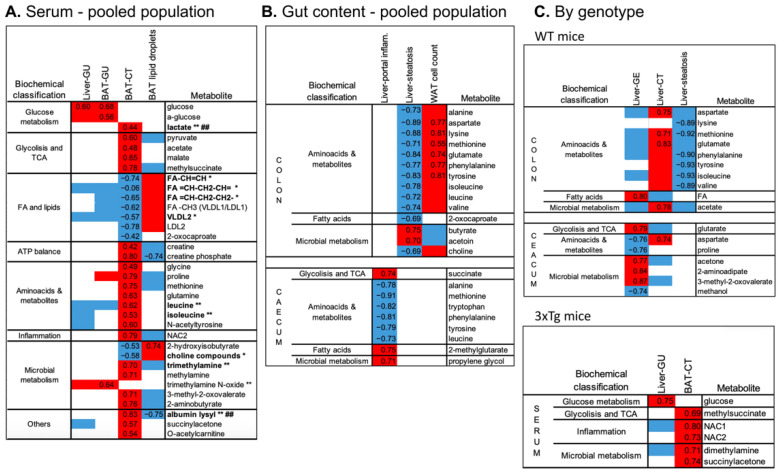
Top significant correlations between imaging and histological parameters and serum (**A**) or colon and caecum fecal metabolome (**B**) assessed in the pooled population (**A**,**B**) or the two genotypes separately (**C**). In the heatmaps, red and blue colors indicate positive and negative correlations, respectively. Spearman’s rho of correlations surviving FDR correction are reported, whereas for univariate correlations not surviving the FDR correction, Spearman’s rho are not reported, but the direction of the association is indicated by the red/blue color. Bold type and symbols are used to highlight metabolites previously shown to be higher (**) or lower (*) in the 3xTg compared to WT mice or shown to be higher (##) in HFD compared to ND fed mice.

Serum metabolome was mainly correlated with BAT radiodensity, showing only a few significant associations with other parameters in the pooled population (Figure 4A). Specifically, higher BAT radiodensity (as observed in 3xTg mice) was significantly related to higher levels of lactate and TCA intermediates (i.e., pyruvate, acetate, maleate, methyl-succinate), creatine and creatine–phosphate, involved in ATP biosynthesis, several amino-acids, including BCAA, and microbial and other products, e.g., TMA and albumin lysis, and to lower levels of fatty acids (saturated, mono- and polyunsaturated), LDL and VLDL. Most of these metabolites (e.g., increased lactate, leucine, and isoleucine, TMA and albumin lysis, or depleted unsaturated fatty acids and VLDL) were previously identified as potential peripheral markers of the 3xTg genotype [22]. Consistently, in the genotype-specific analysis, components of the serum metabolome were related to BAT radiodensity in 3xTg but not in WT mice (Figure 4C).

Among gut metabolites, high amino-acid levels (in the colon) were again correlated with lower lipid accumulation in WAT and liver, as indicated by the direct associations with WAT cell density and the inverse associations with liver steatosis (Figure 4B). High amino-acid levels in the caecum were related to lower liver portal inflammation. Liver fat (steatosis) was also related to colon choline (inversely) and short-chain fatty acid butyrate and acetoin (directly). In the genotype-specific analysis (Figure 4C), colon amino acids were again strongly correlated with lower liver steatosis in WT but not in 3xTg mice. Similarly, components of the caecum metabolome were mainly related to liver metabolism in WT mice only.

### 2.7. Gut Microbial Profile

We have previously identified specific fecal microbiota features associated with the 3xTg genotype and/or with the HFD, including an increased abundance of *Anaeroplasmaptaceae* and *Turicibacteriaceae* in 3xTg mice, an increment in *Rickenellaceae* and *Mogibacteriaceae* in response to HFD, and significant depletion of *Bifidobacteriaceae* and S24.7 in both 3xTg and HFD mice, independent of their genotype [22]. Here, the associations between bacterial abundances and structural and functional parameters of WAT, BAT, and liver were analyzed.

**Figure 5:**
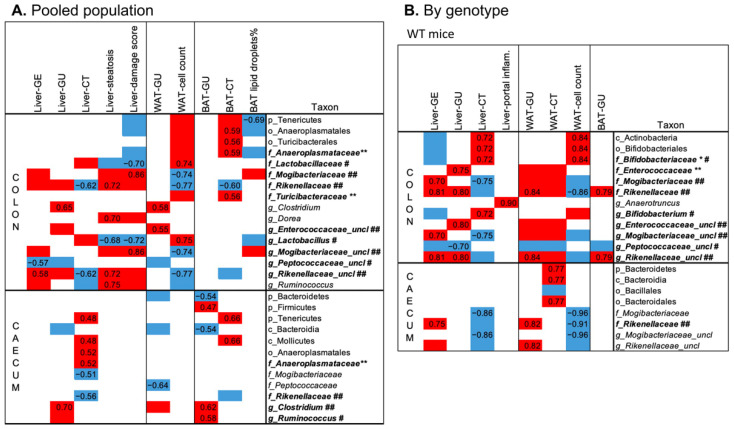
Top significant correlations between imaging and histological parameters and colon and caecum fecal microbiota, assessed in the pooled population (**A**) or the two genotypes separately (**B**). In the heatmaps, red and blue colors indicate positive and negative correlations, respectively. Spearman’s rho of correlations surviving FDR correction are reported, whereas for univariate correlations not surviving the FDR correction, Spearman’s rho are not reported, but the direction of the association is indicated by the red/blue color. Bold type and symbols are used to highlight metabolites previously shown to be higher (**) or lower (*) in the 3xTg compared to WT mice or shown to be higher (##) or lower (#) in HFD compared to ND fed mice.

In the pooled population (Figure 5A), higher colon and caecum abundance of *Anaeroplasmataceae* members (at family, order, class, and phylum levels) predicted greater BAT and liver radiodensity, respectively. BAT radiodensity was also positively related to the abundance of *Turicibacteriaceae*. Greater colon *Rickenellaceae* abundance predicted low BAT radiodensity, low radiodensity, higher steatosis in the liver, and low cell density in WAT. The latter was also positively related to *Lactobacillaceae* (and *Lactobacillus* genus) and inversely to *Mogibacteriaceae* and *Rickenellaceae* abundances in the colon. Liver steatosis was also positively related to colon abundances of *Dorea* and *Ruminococcus* genus (both, Clostridia order, Firmicutes phylum), and inversely to *Lactobacillus* genera. Similar relationships, but with the opposite sign, were observed between the above bacteria and CT radiodensity. Higher abundance of *Mogibacteriaceae* and lower *Lactobacillus* genus abundances in the colon are the main bacterial features of the liver steatoinflammatory score. Fewer correlations were detected between bacteria and organs glucose metabolism. The most relevant is the positive association between *Clostridium* genera (both colon and caecum) and GU of WAT, BAT, and liver.

In WT mice only, higher *Rickenellaceae* and Mogibacteriaceae abundances predicted high liver and WAT lipid content (low radiodensity and cell density, respectively), and high organ GU, whereas higher *Bifidobacterium* abundance (at genus, family, order, class levels) was related to low organ lipid accumulation (Figure 5B). None of these correlations survived in 3xTg mice, in which the microbiota–liver–fat axis appears entirely disrupted.

## 3. Discussion

Study results show that impaired lipid storage in both liver and adipose organs, increased visceral fat GU, and liver inflammation characterized the development and progression of AD cognitive symptoms in the 3xTg mouse model. HFD-induced obesity and aging synergically contributed to the deterioration of the liver, WAT, and BAT metabolic regulation and promoted liver inflammation. These alterations were related to the abundance of specific circulating and gut metabolites and bacteria, and the deterioration of the gut–adipose–tissue–liver–brain axis seems to contribute to the progression of cognitive disease.

Present data confirmed and extended to adipose organs the previously reported defect in liver lipid storage in 3xTg mice fed HFD for 9 months [5], and suggested that 3xTg mice are resistant to fatty liver disease, and BAT whitening. WAT hypermetabolism in adult 3xTg mice might be a compensatory mechanism to prevent lipid storage in other organs. Eventually, WAT cannot further increment its glucose metabolism, which results in a greater glucose exposure of BAT and liver where inflammation is promoted.

Frequent associations were reported between liver pathology, specifically metabolic associated fatty liver disease (MAFLD), and reduced cognitive function [23] or brain activity [24]. However, it is not clear which feature among liver steatosis, dysmetabolism, and liver inflammation plays a major role in the prediction and/or development of cognitive dysfunction [3,4,5]. Our results point out that liver inflammation rather than steatosis is the main culprit. Consistently, liver inflammation and advanced pathological signs of AD were demonstrated in both WT and APP-Tg mice under HFD [4]. Furthermore, elevated circulating ALT levels (here observed in 3xTg mice) were associated with increased amyloid-beta deposition, reduced brain glucose, increased atrophy, and diagnosis of AD in humans [25]. Altogether, these data suggest that liver inflammation may promote neurodegeneration in healthy individuals and accelerate neuro-inflammation and neurodegeneration in genetically predisposed subjects.

The 3xTg model is characterized by a significant peripheral leptin deficiency and shows a lipodystrophic-like phenotype characterized by low bodyweight, smaller adipocytes, and lower lipid content, which is related to low circulating levels of adipose-tissue-derived cytokines, including leptin and MCP-1. Leptin modulates both an innate and adaptive immunity [26], and its deficiency might increase the susceptibility to inflammation [27]. Moreover, leptin has both neurotrophic and neuroprotective effects against AD pathology, as it was shown to decrease Aβ levels consistently [28]. Indeed, lower plasma leptin levels in the late-life of non-obese subjects were related to an increased risk for the development of AD [8] and worse performance in memory tests [29]. In 4- and 12-month-old SAMP8 mice showing elevated beta-amyloid protein and impaired brain function, leptin administration improved memory [30]. In addition, MCP-1 deficiency was related to altered adipose tissue function and metabolism, with the promotion of browning in white and brown adipose tissues [31]. The augmented cellularity at the expense of the lipid content observed in both adipose depots is coherent with an upregulation of the energetic/catabolic metabolism. However, we did not find higher UCP-1 expression in the BAT of 3xTg mice, coherent with BAT thermogenic deficits reported in both transgenic Tg2576 and 3xTg AD mouse models [11,12]. Overall, in 3xTg mice, the lipodystrophy-like alteration to adipose tissues, and the related abnormal cytokine profile, seemed to negatively impact both liver and brain metabolism.

HFD only mildly alleviates the defective lipid storage observed in the 3xTg mice but worsens hepatic inflammation and further increments the already elevated circulating PAI-1, which is positively correlated with portal inflammation and inversely with liver and adipose organs lipids. Consistently in humans, elevated PAI-1 circulating levels, which is mainly related to its liver production [32], were associated with increased ALT and AST levels and correlated with the degree of liver fibrosis in patients with NASH [33]. Furthermore, an increment in plasma PAI-1 was reported in patients with mild cognitive impairment and AD as compared to healthy controls, in proportion to the cognitive function decline [34]. Current evidence indicates that high PAI-1 is related to aβ accumulation [35] and to the impaired maturation of brain-derived neurotrophic factor (BDNF) [36], which is protective for neuronal function and inflammation. Thus, PAI-1 might contribute to the liver–brain axis physio–pathology underlying cognitive diseases.

Chronic INI administration can prevent the observed abnormalities. In adult 3xTg-HFD mice, INI reduced the organs’ GU by lowering HFD-induced glycemia. Moreover, INI increased BAT UCP1 expression, which was shown to be protective in the 3xTg model [12]. With aging, INI promoted the storage of lipids in adipose tissues, increased WAT GU, and reduced liver inflammation. Consistently, rodents and human studies have shown that insulin delivery to the brain suppresses lipolysis [17], increases hepatocellular lipids [18], and stimulates GU into peripheral tissues [37], without modifying peripheral insulin levels. Overall, these results demonstrated that central control of peripheral metabolism can be preserved by an early INI intervention (from 2 months, i.e., before AD symptoms), representing an important avenue to prevent the bidirectional neuronal and adipose tissue co-degeneration seen in 3xTg mice in our study.

The gut microbiota is another promising target to regulate the host metabolism, contributing to the prevention and treatment of cognitive impairment. Published data show that lipid-rich diets can worsen cognitive symptoms [22] and induce brain neuro-inflammation [38], and the effect seems associated with alterations to gut microbes and blood and gut metabolites. Interestingly, in the present study, BAT lipid depletion, e.g., the main hallmark of our 3xTg mice, showed the strongest associations with peculiar circulating and gut metabolome findings, namely lower levels of lipidic species and higher levels of metabolites involved in energy metabolism and ATP biosynthesis. In addition, we found significant correlations between high amino-acids levels, high BAT radiodensity (in the serum), high WAT cell density (in the colon), low liver steatosis, and low portal inflammation (in the colon and caecum, respectively). Branched-chain amino acids (BCAA) are considered protective against hepatic steatosis [39] and the elevated expression of BCAA catabolic genes was shown in BAT [40], suggesting a BCAA-dependent metabolic activation of BAT in 3xTg mice. Furthermore, microbial products were strongly related to BAT lipid content or GU, underscoring the role of a gut–BAT axis in metabolic and cognitive dysfunctions. Deficiency in circulating choline-containing compounds was previously shown in our 3xTg mice [22], and choline deficiency was associated with the disruption of the gut vascular barrier [41], liver inflammation and fibrosis, and low bodyweight [42]. Here, the elevation of choline-derived metabolites trimethylamine (TMA) and trimethylamine N-oxide (TMAO) was related to high BAT radiodensity and GU and low lipid droplets.

Similarly, study results showed significant correlations between organs’ lipid content and the relative abundance of gut bacteria, specifically *Anaeroplasmataceae*, *Lactobacillaceae*, and *Turicibacteriaceae* (inverse), and *Mogibacteriaceae* and *Rikenellaceae* (direct). Interestingly, gut bacteria significantly elevated in the AD genotype, i.e., *Anaeroplasmataceae* and *Turicibacteriaceae*, were correlated with low lipid content in BAT, consistent with the inverse association between the *Anaeroplasma* genus and circulating lipids (IDL-cholesterol, triglycerides) in healthy volunteers [43]. In turn, members of the *Mogibacteriaceae* family, elevated by HFD, were associated with high liver lipid content, total damage score, and GE. The increment in the *Mogibacteriaceae* family abundance was associated with leanness and resistance to diet-induced obesity in a cold exposure experiment [44], and with beta-amyloid levels in the brain of mice suffering from Alzheimer’s disease [45]. Altogether, these data suggested that alterations to the gut microbiota profile might contribute to dysmetabolism and AD progression. In the present study, a higher abundance of the *Lactobacillus* genus was related to low lipid content in both WAT and liver, and a low liver damage score, consistent with a previously reported weight loss when administered to overweight and obese patients [46]. Moreover, the positive association between the *Clostridium* genus and liver, WAT, and BAT GU is consistent with its obesogenic role and the upregulation of small intestinal glucose and fat transporters demonstrated in gnotobiotic mouse models [47]. Finally, it is noteworthy that within-genotype associations were stronger for serum metabolome in 3xTg mice, but not for gut metabolome and microbiota in WT mice, suggesting that gut effects are compromised in 3xTg mice.

The effect of aging resulted in a significant increment in the liver and BAT GU in ND fed mice (irrespective of the strain), and of liver macrovesicular steatosis (in WT only). Conversely, in WT-HFD mice, the detrimental effect of diet was attenuated with aging, as shown by the lack of further increment in GU or lipid accumulation in tissues, the normalization of WAT GU in WT mice, and the reduction in glycemia and triglyceridemia. This finding supports published data reporting that differences between ND and HFD WT mice attenuate with aging due to physiological deterioration of the former and stabilization of the HFD effects in the latter [48]. However, the lack of a longitudinal study design (i.e., different animals were studied at each timepoint) must be acknowledged among the study limitations. A further limitation of the study is the correlative nature of the relationship between metabolic profiles and gut metabolome–microbiota markers, preventing drawing any cause–effect mechanistic conclusion.

In conclusion, study results showed that the 3xTg model of AD was characterized by a generalized (and HFD resistant) deficiency in lipid accumulation in WAT, BAT, and liver, resulting in an abnormal cytokine–hormone profile and liver inflammation, leading to a spiral worsening of both central and peripheral pathology. Hypermetabolism in WAT (at 8 months) and BAT (at 14 months) may limit substrate overflow to other tissues. INI was able to prevent a good part of the adverse neuronal–adipose–tissue co-degenerative cascade. Easily accessible circulating and gut metabolome–microbiota markers predictive of metabolic alterations have been identified, together with the disruption of the gut–microbiota–liver–adipose axis in AD mice. Since an effective pharmacologic treatment is still missing for both liver metabolic disease and AD, the gut microbiota could represent an appealing early target for preventive intervention.

## 4. Materials and Methods

### 4.1. Study Design

The study was conducted in a subset of 88 male mice from the previously published study [2], including 40 controls (B6129SF2/J, strain# 101045) and 48 3xTg mice (B6; 129-Psen1tm1MpmTg(APPSwe,tauP301L)1Lfa/Mmjax; strain#004807, The Jackson Laboratory, Bar Harbor, ME, USA), stratified into 5 groups and studied at age 8 or 14 months: (1) controls normal diet (WT-ND, *n* = 9 + 10, 11% kcals from fat, Mucedola, Milan, Italy); (2) controls high-fat diet (WT-HFD, *n* = 9 + 12, 58% kcals from fat); (3) 3xTg-ND (*n* = 8 + 9); (4) 3xTg-HFD (*n* = 7 + 6); (5) 3xTg-HFD and chronic intranasal insulin therapy (3xTg-HFD + INI, *n* = 11 + 7). The study design has been previously described together with cognitive development and brain metabolism [2]. Here, imaging data and biological samples were re-analyzed, focusing on liver and adipose tissues. Briefly, animals were housed under 12-h light/12-h dark cycles and controlled room temperature (22 °C), with ad libitum access to food and fresh water. Diets and INI were started at 2 months of age. Intranasal insulin (0.87 UL in 24 μL PBS solution (Sigma–Aldrich, St Louis, MO, USA) was administered daily for one week, and weekly thereafter. At 8 and 14 ± 1 months of age, PET-CT imaging with ^18^FDG was performed. At the end of in vivo procedures, animals were euthanized, and the liver, WAT, and interscapular BAT were collected for histological and molecular analyses. In 8-month-old animals, the gut microbiome (colon and caecum) and serum and gut metabolome (colon and caecum) were analyzed. The experimental protocol was conducted under the D.L.116/92 implementation of the European Economic Community directive 609/86 regarding the protection of animals used for experimental and other scientific purposes.

### 4.2. PET-CT Scanning and Image Processing

PET-CT imaging was conducted under fasting conditions in a dedicated µPET-CT tomograph (IRIS PET/CT, Inviscan SAS, Strasbourg, France). Anesthesia was induced by 3–4% (*v*/*v*) and maintained with 1–2% (*v*/*v*) isoflurane. A CT scan was acquired first, then ^18^FDG (7.6 ± 0.1 MBq) was injected i.p., and a 60-min whole-body dynamic PET scan was performed. Glycemia was monitored in tail blood. Body temperature and breath frequency were monitored, and a heated pad was used to prevent the decline in body temperature due to anesthesia.

PET data were corrected for dead time and radioactive decay, reconstructed by standard algorithms and fused to CT images within AMIDE Medical Image Data Examiner 1.0.4. Volumes of interest (VOI) were manually drawn on PET-CT images in the liver, WAT, and BAT. Fractional glucose extraction (GE), reflecting the intrinsic ability of the tissue to actively extract glucose from circulation [49], was expressed as the ratio of tissue activity to the injected ^18^FDG dose per gram of body weight (%ID/g). Glucose uptake (GU) was computed as a product of %ID/g and glycemia during imaging [49]. Tissue radiodensity (Hounsfield unit, Hu) was obtained in the same VOIs from co-registered CT images.

### 4.3. Liver Histology

Liver samples (*n* = 48) were fixed in 10% formalin for 24 h, dehydrated, and included in paraffin using the Donatello Diapath automatic tissue processor (Martinengo, Bergamo, Italy), sliced (HistoCore Autocut, Leica BioSystems microtome) with a thickness of 2 μm, and stained with hematoxylin and eosin, using the automated Dako CoverStainer (Santa Clara, CA, USA). Each section was documented at 20× and 40× magnification, by using the Olympus BX51 microscope connected with an Olympus DP70 digital camera and AnalySIS 5.0 imaging system software (Olympus, Tokyo, Japan). Analyses were adapted from the method of Kleiner et al. [50]. Histological features were grouped into 4 broad categories: microcirculation (portal and central vein, and sinusoidal dilatation), fibrosis (portal, perisinusoidal, perivenular fibrosis), portal inflammation, and lobular damage (micro- and macrovesicular steatosis; lobular inflammation, i.e., foci number, documented at 20×; ballooning degeneration; glycogenated nuclei). Parameters were quantified on a categorical yes/no basis; steatosis was also expressed as percentage of cells affected by microvesicular and/or macrovesicular steatosis (%) and severity grades (0 = ≤5% affected cells, 1 = 6–33% affected cells, 2 = 34–66% affected cells, 3 = ≥67% affected cells), portal inflammation was stratified according to severity grades (1 = minimal, 2 = mild, 3 = moderate), lobular inflammation was stratified according to severity grades, based on foci number at 20× magnification (1 = one focus, 2 = two-four foci, 3 = >four foci), and frequency of ballooning degeneration per field (few cells = 1, many cells = 2). The cumulative steatoinflammatory score was determined by the sum of steatosis grades (1–3), lobular inflammation (1–3), and ballooning grades (1–2), reflecting progressive disease severity stages.

### 4.4. Adipose Tissues Histology

WAT (*n* = 47) and BAT (*n* = 47) samples were fixed in 10% formalin for 24 h, dehydrated, embedded in paraffin (Bio-Optica, Milano, Italy), sliced (Microm HM 330) with a thickness of 5 μm, and stained with hematoxylin and eosin, according to standard protocols. Each section was documented at ×40 magnification using an Axioskop optical microscope connected with an AxioCam MRc5 color-camera and AxioVision analysis software (Carl Zeiss, Oberkochen, Germany). WAT adipocyte number and BAT lipid-laden surface (% of slice area occupancy) were quantified in a minimum of 3 slices in each animal by a blinded operator using the image analysis software ImageJ (version 1.46r, https://imagej.nih.gov/ij). The number of adipocytes per tissue area (cell density) was used as an indirect indicator of intracellular lipid content, i.e., the higher the number of cells per tissue area, the smaller their volume and therefore their lipid content.

### 4.5. BAT UCP1 Protein Expression

In a subset of 8-month-old mice (*n* = 12), UCP1 protein expression was analyzed by Western blot. After dissection, BAT samples were snap-frozen in liquid nitrogen. Protein extraction was performed by adding 100 μL of lysis buffer containing, in mM: 50 Tris-HCL pH 8, 150 NaCl, 2 EDTA, plus 1% NP40, 0.1% SDS, and protease inhibitor cocktails (1:100, P2850, P5726, P8340; Merk Life Science S.r.l., Milano, Italia). After 30 min on ice, the homogenates were centrifuged at 16,000× *g* for 20 min at 4 °C. The supernatants were collected, and the protein concentration of each sample was determined by Bradford Assay (Bio-Rad Laboratories S.r.l., Segrate (Mi), Italia). Then, samples were diluted in 4X sample buffer (Biorad) and boiled at 95 °C for 5 min. For each sample, 40 µg of total protein extract was separated using or 4–20% Criterion TGX precast gels (Bio-Rad Laboratories S.r.l.), and subsequently transferred to nitrocellulose membranes using a semi-dry apparatus (Turbo-Blot, Bio-Rad Laboratories S.r.l.). Membranes were then washed in TBS buffer containing 0.1% Tween-20 (TTBS) and blocked with 5% BSA in TTBS for 1 h at RT. Membranes were incubated O/N at 4 °C with the following primary antibodies: anti UCP-1 (abcam #10983, dil 1:1000), anti-tubulin (1:3000, abcam #52866, dil 1:3000), diluted in TTBS containing 2.5% BSA. After washing in TTBS (3 × 10 min), membranes were reacted with HRP-conjugated goat anti-rabbit secondary antibody (1:20,000, # 1706515, Bio-Rad Laboratories S.r.l.). Signals were revealed using ECL (Biorad). Densitometric analysis was performed using Quantity One software (Bio-Rad Laboratories S.r.l.).

### 4.6. Biochemical Analyses

Triglycerides and liver enzymes (aspartate aminotransferase, AST, alanine aminotransferase, ALT) were determined by a bench clinical chemistry analyzer (Reflovet^®^ Plus, Scil Animal Care Company S.r.l., Treviglio (BG), Italy) at the end of imaging procedures. Triglyceride results falling below the measurable range were equaled to the lowest value of the accessible range (70 mg/dL).

### 4.7. Gut Microbiota Profile by 16S rRNA Amplicon Sequencing

Gut microbial profile was measured in the caecum (*n* = 27) and colon (*n* = 28) content, as previously described [22]. Briefly, total DNA was isolated and measured using a Qubit^®^ 2.0 Fluorometer (Life Technology, Carlsbad, CA, USA). A specific16S rRNA gene fragment (V3–V4 region) was sequenced using a 2 × 300 pb paired-end run (MiSeq Reagent kit v3) on a MiSeq-Illumina platform (FISABIO sequencing service, Valencia, Spain), according to manufacturer’s instructions (Illumina, San Diego, CA, USA). Quality filtering, sequence joining, and chimera removal were obtained using an ad-hoc pipeline written in an RStatistics environment and sequencing processing was performed by QIIME pipeline (version 1.9.0). The clustered sequences were utilized to construct OTUs tables (97% identity), classified into taxonomic levels (Greengenes database), and those tables were used in this study. Sequences not taxonomically classified were removed.

### 4.8. Serum and Fecal Metabolites by 1H-NMR Spectroscopy

Metabolome was analysed in the serum (*n* = 38) and in caecum (*n* = 23) and colon (*n* = 26) content, as previously described [22]. Briefly, serum and fecal extracts (20 μL) supplemented with D2O (2 μL) were transferred into 1-mm NMR tubes. 1H-NMR spectra were recorded in a Bruker Avance DRX 600 spectrometer (Valencia, Spain). Samples were measured at 310 K and a single pulse presaturation experiment was acquired in all samples. Spectra were processed using MestReNova 8.1 (Mestrelab Research S.L., Santiago de Compostela, Spain) and transferred to MATLAB (MathWorks, 2012) using in-house scripts for data analysis. Metabolite spin systems and resonances were identified by literature data and Chenomx resonances database (Chenomx NMR 7.6). Spectra were normalized to the total aliphatic spectral area to eliminate differences in metabolite total concentration, binned into 0.01 ppm buckets, and then subjected to mean-centering. Signals belonging to selected metabolites were integrated and quantified using semi-automated in-house MATLAB peak-fitting routines for obtaining metabolite relative abundances.

### 4.9. Statistical Analysis

IBM^®^ SPSS^®^ Statistics for Mac OS X (version 24.0, Chicago, IL, USA) was used for the statistical analyses of all data, with the exception of microbiota data, for which Calypso software version 8.84 with total sum normalization was used. Imaging, histology, and circulating markers data were analyzed by age groups, and between age groups, and presented as mean ± SEM. Tests for independent samples were used to compare imaging, histology, and circulating markers data, collected in different animals at each age. Most of the data were non-normally distributed according to the Shapiro–Wilk test. Therefore, the Mann–Whitney U test was used for group comparisons, with the exception of BAT lipid droplet %, for which ANOVA with Fisher’s LSD post hoc analysis was used. Spearman’s correlation analysis was used to explore univariate associations between metabolome and microbiota data and organs functional and structural parameters in the population pooled or stratified by genotype. The false discovery rate (FDR) was applied to correct for multiple testing. *p*-values ≤ 0.05 were regarded as statistically significant.

## Figures and Tables

**Table 1:** Bodyweight and circulating markers in study groups.

	*n* (for Each Group)	WT-ND	WT-HFD	3xTg-ND	3xTg-HFD	3xTg-HFD-INI
**8 months**						
Body weight (g)	9-9-8-7-11	41.2 ± 1.4	47.4 ± 2.7 ^	34.5 ± 0.5 ***	37.4 ± 1.6 **,^	30.4 ± 1.2 ###,°°
Glycaemia (mmol/L)	7-9-8-7-11	4.9 ± 0.4	10.4 ± 1.1 ^^^	6.7 ± 0.6	12.6 ± 1.9 ###,***,^^^	7.6 ± 0.3 #,°°
Triglycerides (mg/dL)	9-9-8-6-10	106 ± 11	160 ± 8 ^	94 ± 5	115 ± 19	85 ± 7 #
ALT (U/L)	8-9-7-6-10	72 ± 28	176 ± 37 ^	85 ± 16	53 ± 3 *	39 ± 6
AST (U/L)	8-9-8-6-10	280 ± 159	313 ± 128	678 ± 216 *	667 ± 160 #	657 ± 155 #
Leptin (ng/mL)	8-7-8-7-11	4.7 ± 1.3	6.9 ± 2.4	0.3 ± 0.1 ***	1.8 ± 0.7 ^^	0.9 ± 0.3 ##
Insulin (ng/mL)	7-7-8-7-11	2.9 ± 1.1	4.2 ± 1.2	4.1 ± 1.2	2.3 ± 0.8	1.3 ± 0.2
IL-6 (pg/mL)	9-9-8-6-10	124 ± 37	114 ± 23	63 ± 13	62 ± 21 *	148 ± 37 °
MCP-1 (pg/mL)	9-9-8-7-10	40 ± 11	37 ± 10	16 ± 2 **	22 ± 5 *	41 ± 8 °
PAI-1 (ng/mL)	9-8-6-7-10	5.5 ± 1.2	6.1 ± 1.4	10.0 ± 1.2 *	17.7 ± 3.9 ###,**	14.6 ± 3.3 ##
Resistin (pg/mL)	9-9-8-7-11	278 ± 87	346 ± 68	180 ± 40	374 ± 30 ^	382 ± 69
**14 months**						
Body weight (g)	10-12-9-6-7	51.3 ± 1.9 $	58.5 ± 2.6 ^,$	38.7 ± 0.8 ***,$	51.2 ± 6.8 ^^	41.2 ± 3.9 #,°,$
Glucose (mmol/L)	10-12-8-6-7	7.4 ± 1.2	6.9 ± 0.7 $	8.3 ± 0.6	7.9 ± 2.2 $	11.2 ± 1.4 #
Triglycerides (md/dL)	9-11-9-5-7	95 ± 9	91 ± 6 $	73 ± 3 *,$	79 ± 6 $	87 ± 10
ALT (U/L)	7-11-8-5-6	110 ± 28	138 ± 25	51 ± 10	192 ± 98	71 ± 13
AST (U/L)	9-11-8-3-7	578 ± 133	560 ± 131	485 ± 92	131 ± 25	480 ± 88
Leptin (ng/mL)	10-11-9-6-7	14.4 ± 2.4 $	20.7 ± 3.1 $	0.5 ± 0.1 ***,$	2.7 ± 0.9 ###,***,^	2.3 ± 0.9 ###,$
Insulin (ng/mL)	10-11-9-6-7	3.8 ± 0.8	10.1 ± 2.2	2.9 ± 0.8	3.1 ± 1.2 *	2.5 ± 1.1
IL-6 (pg/mL)	8-10-8-6-7	1618 ± 472	1247 ± 571 $	145 ± 90 ***	606 ± 318 #	311 ± 229 ###
MCP-1 (pg/mL)	9-10-9-6-7	97 ± 22 $	71 ± 16 $	45 ± 24 *	35 ± 11 #	20 ± 2 ##
PAI-1 (ng/mL)	10-12-9-5-7	9.8 ± 2.4	12.6 ± 2.9	15.7 ± 3.8 *	16.8 ± 2.7	10.4 ± 2.0
Resistin (pg/mL)	10-12-9-6-7	874 ± 244 $	957 ± 127 $	279 ± 124 *	854 ± 209 ^^	296 ± 58 °

Legend: different symbols are used for each within-age comparison, * *p* ≤ 0.05 vs. WT, ** *p* ≤ 0.01 vs. WT, *** *p* ≤ 0.001 vs. WT, ^ *p* ≤ 0.05 vs. ND, ^^ *p* ≤ 0.01 vs. ND, ^^^ *p* ≤ 0.001 vs. ND, # *p* ≤ 0.05 vs. WT-ND, ## *p* ≤ 0.01 vs. WT-ND, ### *p* ≤ 0.001 vs. WT-ND, ° *p* ≤ 0.05 vs. 3xTg-HFD, °° *p* ≤ 0.01 vs. 3xTg-HFD. The symbol $ is used to identify statistically significant differences in between-age comparisons.

**Table 2:** Correlations between circulating markers and tissue structure and functional parameters.

	8 Months			14 Months						
	TG (mg/dL)	Leptin (pg/mL)	PAI-1 (pg/mL)	TG (mg/dL)	Leptin (pg/mL)	PAI-1 (pg/mL)	IL6 (pg/mL)	MCP-1 (pg/mL)	Insulin (pg/mL)	Resistin (pg/mL)
Liver GE	0.271	−0.061	0.172	0.304	0.409 **	−0.112	0.478 **	0.471 **	0.278	0.542 **
Liver GU	0.493 **	0.067	0.174	0.315 *	0.239	−0.295	0.035	0.192	−0.051	0.100
Liver radiodensity	−0.436 **	−0.598 **	0.199	−0.577 **	−0.885 **	0.321 *	−0.420 *	−0.615 **	−0.507 **	−0.500 **
Liver macrovescicular steatosis	0.558 **	0.496 *	−0.037	0.364	0.601 **	−0.505 *	0.263	0.469 *	0.131	0.343
Liver total steatosis	0.550 **	0.578 **	−0.187	0.437 *	0.560 **	−0.588 **	0.050	0.080	0.008	0.189
Liver portal inflammation	−0.518 *	−0.433 *	0.489 *	−0.314	−0.449 *	0.347	−0.260	−0.386	−0.497 *	−0.402
Liver ballooning	0.649 **	0.405	−0.285	0.008	0.363	−0.272	0.214	0.398	0.212	0.333
Liver steatoinflammatory score	0.522 *	0.590 **	−0.264	0.232	0.370	−0.369	−0.058	0.422 *	−0.048	0.185
WAT radiodensity	−0.530 **	−0.536 **	0.350 *	−0.516 **	−0.781 **	0.243	−0.545 **	−0.604 **	−0.394 *	−0.436 **
WAT cell count	−0.634 **	−0.700 **	0.223	−0.323	−0.493 *	0.579 **	−0.205	−0.272	−0.013	−0.136
BAT radiodensity	−0.603 **	−0.758 **	0.437 **	−0.582 **	−0.822 **	0.354 *	−0.468 **	−0.546 **	−0.506 **	−0.452 **
BAT lipid droplets	0.622 **	0.643 **	−0.382	0.614 **	0.647 **	−0.559 **	0.474 *	0.369	0.245	0.522 **

Legend: the table reports Spearman’s rho, * *p* < 0.05, ** *p* < 0.01.

## Data Availability

The data presented in this study are available on request from the corresponding author, as they have not yet been uploaded in a public database.
